# Supplementary material for: Serum Uric Acid Predicts All-Cause and Cardiovascular Mortality Independently of Hypertriglyceridemia in Cardiometabolic Patients without Established CV Disease: A Sub-Analysis of the URic acid Right for heArt Health (URRAH) Study
Source: Metabolites. 2023 Feb 7;13(2):244. doi: 10.3390/metabo13020244 (PMC9959524; doi:10.3390/metabo13020244)
Supplement: Supplementary file 1 [file metabolites-13-00244-s001.zip › metabolites-2205686-supplementary.pdf]

SUPPLEMENTARY TABLES AND SUPPLEMENTARY FIGURE

Serum uric acid predicts all-cause and cardiovascular mortality independently of hypertriglyceridemia in cardiometabolic patients without established CV disease: a sub-analysis of the URic acid Right for heArt Health (URRAH) study

Alessandro Mengozzi [1,2,3]*, Nicola Riccardo Pugliese [1]*, Stefano Masi [1], Fabio Angeli [4], Carlo M. Barbagallo [5], Michele Bombelli [6], Federica Cappelli [1], Edoardo Casiglia [7], Rosario Cianci [8], Michele Ciccarelli [9], Arrigo F.G. Cicero [10,11], Massimo Cirillo [12], Pietro Cirillo [13], Raffaella Dell'Oro [6], Lanfranco D'Elia [14], Giovambattista Desideri [15], Claudio Ferri [15], Ferruccio Galletti [14], Loreto Gesualdo [13], Cristina Giannattasio [16,17], Guido Grassi [6], Guido Iaccarino [9], Luciano Lippa [18], Francesca Mallamaci [19], Alessandro Maloberti [16,17], Maria Masulli [14], Alberto Mazza [20], Maria Lorenza Muiesan [21], Pietro Nazzaro [22], Paolo Palatini [7], Gianfranco Parati [23], Roberto Pontremoli [24], Fosca Quarti-Trevano [6], Marcello Rattazzi [25], Gianpaolo Reboldi [26], Giulia Rivasi [27], Elisa Russo [24], Massimo Salvetti [21], Valerie Tikhonoff [28], Giuliano Tocci [29], Andrea Ungar [27], Paolo Verdecchia [30], Francesca Viazzi [24], Massimo Volpe [29], Claudio Borghi [10,11]*, Agostino Virdis [1]* on behalf of the Working Group on Uric Acid and Cardiovascular Risk of the Italian Society of Hypertension (SIIA)

[1] Department of Clinical and Experimental Medicine, University of Pisa, Italy
[2] Center for Translational and Experimental Cardiology (CTEC), Department of Cardiology, University Hospital Zurich, University of Zurich, Switzerland.
[3] Scuola Superiore Sant'Anna, Pisa, Italy
[4] Department of Medicine and Surgery. University of Insubria, Varese, and Department of Medicine and Cardiopulmonary Rehabilitation. Maugeri Care and Research Institutes, IRCCS Tradate, Varese, Italy
[5] Biomedical Department of Internal Medicine and Specialistics, University of Palermo, Palermo, Italy
[6] Clinica Medica, Department of Medicine and Surgery, University of Milano-Bicocca, Monza, Italy
[7] Studium Patavinum, Department of Medicine, University of Padua, Padua, Italy
[8] Department of Translational and Precision Medicine, Sapienza University of Rome, Rome
[9] Department of Advanced Biomedical Sciences, ''Federico II'' University of Naples, Naples, Italy
[10] Department Hypertension and Cardiovascular Disease Research Center, Medical and Surgical Sciences Dept., Alma Mater Studiorum University of Bologna, Bologna, Italy
[11] Heart-Chest-Vascular Dept., IRCCS AOU of Bologna, Bologna, Italy

[12] Department of Public Health, "Federico II" University of Naples, Naples, Italy
[13] Nephrology, Dialysis and Transplantation Unit, Department of Emergency and Organ Transplantation, ''Aldo Moro'' University of Bari, Bari, Italy
[14] Department of Clinical Medicine and Surgery, 'Federico II' University of Naples, Naples, Italy
[15] Department of Life, Health and Environmental Sciences, University of L'Aquila, L'Aquila, Italy
[16] Cardiology IV, ''A.De Gasperi's'' Department, Niguarda Ca' Granda Hospital, Milan, Italy
[17] School of Medicine and Surgery, Milano-Bicocca University, Milan, Italy
[18] Italian Society of General Medicine (SIMG), Avezzano, L'Aquila, Italy
[19] CNR-IFC, Clinical Epidemiology of Renal Diseases and Hypertension, Reggio Cal Unit, Reggio Calabria, Italy
[20] Department of Internal Medicine, Santa Maria Della Misericordia General Hospital, AULSS 5 Polesana, Rovigo, Italy
[21] Department of Clinical and Experimental Sciences, University of Brescia, Italy
[22] Department of Precision and Regenerative Medicine and Jonic Area (DiMePRe-J), Neurosciences and Sense Organs, University of Bari Medical School, Bari, Italy
[23] S. Luca Hospital, Istituto Auxologico Italiano & University of Milan-Bicocca, Milan, Italy
[24] Department of Internal Medicine, University of Genoa; IRCSS Ospedale Policlinico San Martino, Genova, Italy
[25] Department of Medicine - DIMED, University of Padova, Medicina Interna 1°, Ca' Foncello University Hospital, Treviso, Italy
[26] Department of Medical and Surgical Science, University of Perugia, Perugia, Italy
[27] Department of Geriatric and Intensive Care Medicine, Careggi Hospital and University of Florence, Italy
[28] Department of Medicine, University of Padua, Padua, Italy
[29] Department of Clinical and Molecular Medicine, University of Rome Sapienza, Rome, Italy
[30] Hospital S. Maria della Misericordia, Perugia, Italy

* Alessandro Mengozzi and Nicola Riccardo Pugliese joint first authorship.

Claudio Borghi and Agostino Virdis joint last authorship.

Corresponding author:

Alessandro Mengozzi, MD

Department of Clinical and Experimental Medicine, University of Pisa, Italy

Via Savi 10

Email: alessandro.mengozzi@medmcs.unipi.it

Fax +39 050 995728

Tel +39 050 992558

Supplementary Table S1. Exploration of all-cause mortality across the cardiometabolic spectrum for SUA≥4.7 mg/dL.

**All-cause mortality**				
			Univariable	Multivariable
Healthy (n=1956)				
	hTG	n=231	2.35 [0.91-6.08], p=0.079	2.35 [0.84-6.55], p=0.102
	nTG	n=1725	1.99 [1.47-2.69], p<0.001	1.55 [1.09-2.21], p<0.015
Obese (n=148)				
	hTG	n=40	>100 [0.00->100], p=0.999	>100 [0.00->100], p=0.979
	nTG	n=108	3.98 [1.16-13.69], p=0.028	5.79 [1.20-27.98], p=0.029
Hypertensive (n=4227)				
	hTG	n=961	1.41 [1.01-1.96], p=0.043	0.96 [0.67-1.38], p=0.840
	nTG	n=3266	1.70 [1.45-1.98], p<0.001	1.28 [1.09-1.52]. p=0.002
Diabetic (n=75)				
	hTG	n=19	1.53 [0.41-5.68], p=0.523	2 [1.06->100]; p<0.001
	nTG	n=56	1.66 [0.81-3.37], p=0.164	1.21 [0.49-3.00]. p=0.677
Obese and hypertensive (n=919)				
	hTG	n=317	1.93 [0.83-4.47], p=0.126	1.31 [0.55-3.13], p=0.536
	nTG	n=602	1.13 [0.75-1.71], p=0.564	0.86 [0.55-1.35], p=0.520
Obese and diabetic (n=18)				
	hTG	n=9	n/a	n/a
	nTG	n=9	n/a	n/a
Hypertensive and diabetic (n=524)				
	hTG	n=178	1.63 [0.98-2.69], p=0.058	1.13 [0.65-1.98], p=0.669
	nTG	n=346	1.66 [1.19-2.32], p=0.003	1.36 [0.96-2.01], p=0.082
Obese, hypertensive and diabetic (n=257)				
	hTG	n=110	2.70 [0.97-7.53], p=0.057	2.70 [0.91-7.98], p=0.072
	nTG	n=147	1.19 [0.68-2.08], p=0.533	1.28 [0.67-2.46], p=0.452

Hazard ratios [95% confidence interval] for SUA cut-off across TG strata in univariable and multivariable analysis for all-cause mortality across the cardiometabolic spectrum. Age, sex, BMI, the active consumption of alcohol, the current smoking habit, SBP, DBP, total cholesterol, HDL, glycaemia, creatinine, hematocrit and diuretics were used as confounders. Data were analysed by Cox regression analysis. The analysis was not performed on people with obesity and type diabetes and no other comorbidities due to the small sample size of the group (n=18, n=9 for nTG and hTG subgroups). Data are reported for completeness in undersized groups (n<50). p <0.05 was considered statistically significant *>100: hazard ratio or confidence interval value higher than 100. nTG: normotriglyceridemia. hTG: hypertriglyceridemia. SUA: serum uric acid.*

Supplementary Table S2. Exploration of cardiovascular mortality across the cardiometabolic spectrum for SUA≥5.6 mg/dL.

**Cardiovascular mortality**				
			Univariable	Multivariable
Healthy (n=1956)				
	hTG	n=231	1.56 [0.45-5.39], p=0.482	3.80 [0.76-19.10], p=0.105
	nTG	n=1725	1.89 [1.14-3.16], p=0.014	1.46 [0.83-2.68], p=0.188
Obese (n=148)				
	hTG	n=40	>100 [0.00->100], p=0.999	50.94 [0.00->100], p=1.000
	nTG	n=108	4.52 [1.08-18.98], p=0.039	5.08 [0.78-43.83], p=0.085
Hypertensive (n=4227)				
	hTG	n=961	1.57 [1.07-2.31], p=0.021	1.19 [0.77-1.84], p=0.442
	nTG	n=3266	1.90 [1.53-2.37], p<0.001	1.37 [1.08-1.74], p=0.010
Diabetic (n=75)				
	hTG	n=19	2.32 [0.49-10.91], p=0.286	0.00 [0->100], p=0.993
	nTG	n=56	3.82 [1.46-10-05], p=0.007	6.92 [1.50-31.91], p=0.013
Obese and hypertensive (n=919)				
	hTG	n=313	2.01 [0.85-4.76], p=0.112	1.50 [0.58-3.93], p=0.404
	nTG	n=602	2.08 [1.19-3.62], p=0.010	1.86 [1.00-3.45], p=0.051
Obese and diabetic (n=18)				
	hTG	n=9	n/a	n/a
	nTG	n=9	n/a	n/a
Hypertensive and diabetic (n=524)				
	hTG	n=178	1.76 [1.00-3.12], p=0.051	1.39 [0.75-2.59], p=0.298
	nTG	n=346	1.92 [1.29-2.95], p=0.002	1.60 [1.02-2.53], p=0.043
Obese, hypertensive and diabetic (n=257)				
	hTG	n=110	1.45 [0.66-3.19], p=0.360	1.46 [0.58-3.68], p=0.426
	nTG	n=147	1.00 [0.53-1.90], p=0.989	1.12 [0.55-2.58], p=0.752

Hazard ratios [95% confidence interval] for SUA cut-off across TG strata in univariable and multivariable analysis for cardiovascular and mortality across the cardiometabolic spectrum. Age, sex, BMI, Age, sex, BMI, the active consumption of alcohol, the current smoking habit, SBP, DBP, total cholesterol, HDL, glycaemia, creatinine, hematocrit and diuretics were used as confounders. Data were analysed by Cox regression analysis. The analysis was not performed on people with obesity and type diabetes and no other comorbidities due to the small sample size of the group (n=18, n=9 for nTG and hTG subgroups). Data are reported for completeness in undersized groups (n<50). p <0.05 was considered statistically significant *>100: hazard ratio or confidence interval value higher than 100. nTG: normotriglyceridemia. hTG: hypertriglyceridemia. SUA: serum uric acid.*

Supplementary Table S3. Exploration of all-cause mortality across the cardiometabolic spectrum of combined SUA and TG strata adopting SUA≥4.7 mg/dL and TG≥150 mg/dL as cut-offs.

**All-cause mortality**				
			Univariable	Multivariable
Healthy (n=1956)				
	lSUA_nTG	n=985	1 (reference)	1 (reference)
	hSUA_nTG	n=740	1.99 [1.47-2.69], p<0.001	1.55 [1.10-2.18], p=0.012
	lSUA_hTG	n=74	0.96 [0.39-2.37], p=0.926	0.60 [0.23-1.53], p=0.281
	hSUA_hTG	n=157	2.36 [1.53-2.69], p<0.001	1.71 [1.05-2.81], p=0.033
Obese (n=148)				
	lSUA_nTG	n=42	1 (reference)	1 (reference)
	hSUA_nTG	n=66	3.90 [1.14-13.41], p=0.031	5.64 [1.24-25.59, p=0.025
	lSUA_hTG	n=7	0.00 [0.00->100], p=0.997	0.00 [0.00->100], p=0.998
	hSUA_hTG	n=33	2.36 [0.59-9.43], p=0.225	6.29 [0.98-40.29], p=0.052
Hypertensive (n=4227)				
	lSUA_nTG	n=1486	1 (reference)	1 (reference)
	hSUA_nTG	n=1780	1.69 [1.45-1.98], p<0.001	1.25 [1.06-1.47], p=0.009
	lSUA_hTG	n=237	1.07 [0.78-1.47], p=0.682	1.45 [1.04-2.03], p=0.029
	hSUA_hTG	n=724	1.52 [1.25-1.84], p<0.001	1.61 [1.29-2.01], p<0.001
Diabetic (n=75)				
	lSUA_nTG	n=25	1 (reference)	1 (reference)
	hSUA_nTG	n=31	1.64 [0.81-3.34], p=0.171	1.33 [0.56-3.15], p=0.521
	lSUA_hTG	n=5	1.14 [0.32-4.03], p=0.844	2.34 [0.50-10.91], p=0.278
	hSUA_hTG	n=14	1.92 [0.80-4.60], p=0.141	1.66 [0.54-5.10], p=0.375
Obese and hypertensive (n=919)				
	lSUA_nTG	n=182	1 (reference)	1 (reference)
	hSUA_nTG	n=420	1.13 [0.74-1.71], p=0.571	0.95 [0.61-1.47], p=0.810
	lSUA_hTG	n=261	0.69 [0.29-1.66], p=0.409	0.99 [0.40-2.44], p=0.981
	hSUA_hTG	n=56	1.35 [0.87-2.09], p=0.179	1.27 [0.77-2.12], p=0.352
Obese and diabetic (n=18)				
	lSUA_nTG	n=3	1 (reference)	1 (reference)
	hSUA_nTG	n=6	n/a	n/a
	lSUA_hTG	n=5	n/a	n/a
	hSUA_hTG	n=4	n/a	n/a
Hypertensive and diabetic (n=524)				
	lSUA_nTG	n=137	1 (reference)	1 (reference)
	hSUA_nTG	n=209	1.66 [1.19-2.32], p=0.003	1.40 [0.98-2.00], p=0.065
	lSUA_hTG	n=56	1.05 [0.63-1.77], p=0.843	1.43 [0.82-2.50], p=0.210
	hSUA_hTG	n=122	1.72 [1.19-2.48], p=0.004	1.83 [1.19-2.82], p=0.006
Obese, hypertensive and diabetic (n=257)				
	lSUA_nTG	n=42	1 (reference)	1 (reference)
	hSUA_nTG	n=105	1.19 [0.68-2.08], p=0.537	1.05 [0.58-1.90], p=0.874
	lSUA_hTG	n=19	0.52 [0.17-1.54], p=0.234	0.35 [0.11-1.11], p=0.075
	hSUA_hTG	n=91	1.46 [0.83-2.56], p=0.188	1.19 [0.63-2.20], p=0.613

Hazard ratios [95% confidence interval] for SUA and TG cut-offs in univariable and multivariable analysis for all-cause mortality across the cardiometabolic spectrum. Age, sex, BMI, the active consumption of alcohol, the current smoking habit, SBP, DBP, total cholesterol, HDL, glycaemia, creatinine, hematocrit and diuretics were used as confounders. Data were analysed by Cox regression analysis. The analysis was not performed on people with obesity and type diabetes and no other comorbidities due to the small sample size of the group (n=18). Data are reported for completeness in undersized groups (n<50). $p < 0.05$ was considered statistically significant *>100: hazard ratio or confidence interval value higher than 100. lSUA_hTG: low SUA and hypertriglyceridemia TG. lSUA_nTG: low SUA and normotriglyceridemia. hSUA_hTG: high SUA and hypertriglyceridemia TG. hSUA_nTG: high SUA and normotriglyceridemia. TG: triglycerides. SUA: serum uric acid*

Supplementary Table S4. Exploration of cardiovascular mortality across the cardiometabolic spectrum of combined SUA and TG strata adopting SUA≥5.6 mg/dL and TG≥150 mg/dL as cut-offs.

**Cardiovascular mortality**				
			Univariable	Multivariable
Healthy (n=1956)				
	lSUA_nTG	n=1410	1 (reference)	1 (reference)
	hSUA_nTG	n=315	1.90 [1.14-3.16], p=0.014	1.48 [0.85-2.59], p=0.167
	lSUA_hTG	n=144	0.99 [0.40-2.49], p=0.988	0.79 [0.30-2.13], p=0.648
	hSUA_hTG	n=87	1.57 [0.63-3.94], p=0.336	0.93 [0.34-2.58], p=0.890
Obese (n=148)				
	lSUA_nTG	n=77	1 (reference)	1 (reference)
	hSUA_nTG	n=31	4.48 [1.07-18.79], p=0.040	3.67 [0.70-19.29], p=0.124
	lSUA_hTG	n=17	0.00 [0.00->100], p=0.998	0.00 [0.00->100], p=0.999
	hSUA_hTG	n=23	1.99 [0.33-11.94], p=0.450	2.70 [0.27-27.26], p=0.399
Hypertensive (n=4227)				
	lSUA_nTG	n=2354	1 (reference)	1 (reference)
	hSUA_nTG	n=912	1.90 [1.53-2.37], p<0.001	1.35 [1.07-1.71], p=0.012
	lSUA_hTG	n=501	1.06 [0.77-1.46], p=0.721	1.29 [0.91-1.82], p=0.154
	hSUA_hTG	n=460	1.67 [1.25-2.23], p<0.001	1.62 [1.17-2.25], p=0.004
Diabetic (n=75)				
	lSUA_nTG	n=35	1 (reference)	1 (reference)
	hSUA_nTG	n=21	3.67 [1.41-9.59], p=0.008	4.36 [1.20-15.80], p=0.025
	lSUA_hTG	n=11	1.48 [0.38-5.73], p=0.569	14.05 [1.94->100], p=0.009
	hSUA_hTG	n=8	4.83 [1.35-17.22], p=0.015	4.10 [0.76-22-16], p=0.101
Obese and hypertensive (n=919)				
	lSUA_nTG	n=335	1 (reference)	1 (reference)
	hSUA_nTG	n=247	2.07 [1.19-3.60], p=0.010	1.96 [1.09-3.53], p=0.025
	lSUA_hTG	n=128	0.93 [0.40-2.18], p=0.870	1.58 [0.64-3.89], p=0.318
	hSUA_hTG	n=189	1.88 [1.02-3.44], p=0.042	2.45 [1.17-5.10], p=0.017
Obese and diabetic (n=18)				
	lSUA_nTG	n=4	1 (reference)	1 (reference)
	hSUA_nTG	n=5	n/a	n/a
	lSUA_hTG	n=6	n/a	n/a
	hSUA_hTG	n=3	n/a	n/a
Hypertensive and diabetic (n=524)				
	lSUA_nTG	n=223	1 (reference)	1 (reference)
	hSUA_nTG	n=123	1.94 [1.28-2.94], p=0.002	1.65 [1.07-2.57], p=0.025
	lSUA_hTG	n=56	1.07 [0.63-1.80], p=0.812	1.31 [0.74-2.33], p=0.359
	hSUA_hTG	n=122	1.87 [1.18-2.96], p=0.008	1.78 [1.04-3.05], p=0.034
Obese, hypertensive and diabetic (n=257)				
	lSUA_nTG	n=82	1 (reference)	1 (reference)
	hSUA_nTG	n=65	1.00 [0.53-1.90], p=0.993	0.98 [0.50-1.94], p=0.955
	lSUA_hTG	n=47	0.82 [0.39-1.74], p=0.606	0.72 [0.32-1.63], p=0.435
	hSUA_hTG	n=63	1.16 [0.61-2.23], p=0.652	1.11 [0.53-2.32], p=0.783

Hazard ratios [95% confidence interval] for SUA and TG cut-offs in univariable and multivariable analysis for cardiovascular mortality across the cardiometabolic spectrum. Age, sex, BMI, the active consumption of alcohol, the current smoking habit, SBP, DBP, total cholesterol, HDL, glycaemia, creatinine, hematocrit and diuretics were used as confounders. Data were analysed by Cox regression analysis. The analysis was not performed on people with obesity and type diabetes and no other comorbidities due to the small sample size of the group (n=18). Data are reported for completeness in undersized groups (n<50). p <0.05 was considered statistically significant >*100: hazard ratio or confidence interval value higher than 100. lSUA_hTG: low SUA and hypertriglyceridemia TG. lSUA_nTG: low SUA and normotriglyceridemia. hSUA_hTG: high SUA and hypertriglyceridemia TG. hSUA_nTG: high SUA and normotriglyceridemia. TG: triglycerides. SUA: serum uric acid.*

Supplementary Table S5. Exploration of all-cause mortality across the cardiometabolic spectrum for SUA/serum creatinine>5.35.

**All-cause mortality**				
			Univariable	Multivariable
Healthy (n=1956)				
	hTG	n=231	2.08 [0.90-4.80], p=0.086	2.50 [1.02-6.14], p=0.045
	nTG	n=1725	1.19 [0.89-1.60], p=0.239	1.23 [0.90-1.67], p=0.189
Obese (n=148)				
	hTG	n=40	>100 [0.00->100], p=0.999	>100 [0.00->100], p=0.998
	nTG	n=108	1.58 [0.62-4.03], p=0.334	4.24 [1.22-14.74], p=0.023
Hypertensive (n=4227)				
	hTG	n=961	1.07 [0.81-1.42], p=0.614	0.89 [0.67-1.19], p=0.443
	nTG	n=3266	1.40 [1.21-1.62], p<0.001	1.23 [1.06-1.44], p=0.006
Diabetic (n=75)				
	hTG	n=19	1.78 [0.53-5.94], p=0.348	10.92 [0.12-999.26], p=0.300
	nTG	n=56	1.30 [0.65-2.62], p=0.462	1.03 [0.39-2.70], p=0.958
Obese and hypertensive (n=919)				
	hTG	n=317	0.93 [0.53-1.64], p=0.170	1.80 [1.01-3.21], p=0.046
	nTG	n=602	1.79 [1.14-2.82], p=0.012	1.31 [0.82-2.08], p=0.252
Obese and diabetic (n=18)				
	hTG	n=9	n/a	n/a
	nTG	n=9	n/a	n/a
Hypertensive and diabetic (n=524)				
	hTG	n=178	1.02 [0.66-1.57], p=0.922	1.06 [0.65-1.71], p=0.829
	nTG	n=346	1.11 [0.81-1.51], p=0.530	1.08 [0.77-1.51], p=0.665
Obese, hypertensive and diabetic (n=257)				
	hTG	n=110	1.11 [0.58-2.09], p=0.756	1.16 [0.59-2.30], p=0.667
	nTG	n=147	1.34 [0.79-2.26], p=0.281	1.67 [0.92-3.04], p=0.093

Hazard ratios [95% confidence interval] for SUA/serum creatinine cut-off across TG strata in univariable and multivariable analysis for all-cause mortality across the cardiometabolic spectrum. Age, sex, BMI, the active consumption of alcohol, the current smoking habit, SBP, DBP, total cholesterol, HDL, glycaemia, hematocrit and diuretics were used as confounders. Data were analysed by Cox regression analysis. The analysis was not performed on people with obesity and type diabetes and no other comorbidities due to the small sample size of the group (n=18, n=9 for nTG and hTG subgroups). Data are reported for completeness in undersized groups (n<50). p <0.05 was considered statistically significant *>100: hazard ratio or confidence interval value higher than 100. nTG: normotriglyceridemia. hTG: hypertriglyceridemia. SUA: serum uric acid.*

Supplementary Table S6. Exploration of cardiovascular mortality across the cardiometabolic spectrum for SUA/serum creatinine>5.35.

**Cardiovascular mortality**				
			Univariable	Multivariable
Healthy (n=1956)				
	hTG	n=231	4.99 [0.63-39.44], p=0.811	10.48 [1.10-99.60], p=0.041
	nTG	n=1725	1.29 [0.81-2.07], p=0.285	1.41 [0.85-2.33], p=0.183
Obese (n=148)				
	hTG	n=40	>100 [0.00->100], p=0.999	>100 [0.00->100], p=0.979
	nTG	n=108	2.58 [0.52-12.81], p=0.246	16.23 [1.03-256.66], p=0.048
Hypertensive (n=4227)				
	hTG	n=961	1.05 [0.71-1.56], p=0.791	0.83 [0.55-1.26], p=0.389
	nTG	n=3266	1.40 [1.13-1.74], p=0.002	1.19 [0.95-1.48], p=0.122
Diabetic (n=75)				
	hTG	n=19	1.19 [0.26-5.35], p=0.822	>100 [0->100], p=0.999
	nTG	n=56	1.12 [0.44-2.85], p=0.815	0.62 0.14-2.67], p=0.520
Obese and hypertensive (n=919)				
	hTG	n=317	1.10 [0.45-2.74], p=0.830	1.09 [0.43-2.79], p=0.849
	nTG	n=602	1.84 [0.94-3.59], p=0.073	1.20 [0.60-2.40], p=0.607
Obese and diabetic (n=18)				
	hTG	n=9	n/a	n/a
	nTG	n=9	n/a	n/a
Hypertensive and diabetic (n=524)				
	hTG	n=178	1.19 [0.67-2.12], p=0.544	1.25 [0.64-2.42], p=0.512
	nTG	n=346	1.24 [0.82-1.88], p=0.314	1.18 [0.75-1.84], p=0.477
Obese, hypertensive and diabetic (n=257)				
	hTG	n=110	0.93 [0.40-2.14], p=0.861	1.14 [0.44-2.93], p=0.794
	nTG	n=147	1.43 [0.72-2.84], p=0.307	2.45 [1.08-5.57], p=0.032

Hazard ratios [95% confidence interval] for SUA/serum creatinine cut-off across TG strata in univariable and multivariable analysis for cardiovascular mortality across the cardiometabolic spectrum. Age, sex, BMI, the active consumption of alcohol, the current smoking habit, SBP, DBP, total cholesterol, HDL, glycaemia, hematocrit and diuretics were used as confounders. Data were analysed by Cox regression analysis. The analysis was not performed on people with obesity and type diabetes and no other comorbidities due to the small sample size of the group (n=18, n=9 for nTG and hTG subgroups). Data are reported for completeness in undersized groups (n<50). p <0.05 was considered statistically significant *>100: hazard ratio or confidence interval value higher than 100. nTG: normotriglyceridemia. hTG: hypertriglyceridemia. SUA: serum uric acid.*

Supplementary Table S7. Exploration of all-cause mortality across the cardiometabolic spectrum for combined SUA and TG strata adopting SUA/serum creatinine>5.35 and TG≥150 mg/dL as cut-offs.

**All-cause mortality**				
			Univariable	Multivariable
Healthy (n=1956)				
	lSUA/sCR_nTG	n=863	1 (reference)	1 (reference)
	hSUA/sCR_nTG	n=862	1.19 [0.89-1.60], p=0.238	1.20 [0.89-1.63], p=0.232
	lSUA/sCR_hTG	n=151	0.85 [0.39-1.84], p=0.681	0.60 [0.27-1.33], p=0.206
	hSUA/sCR_hTG	n=80	1.85 [1.19-2.86], p=0.006	1.43 [0.89-2.30], p=0.137
Obese (n=148)				
	lSUA/sCR_nTG	n=44	1 (reference)	1 (reference)
	hSUA/sCR_nTG	n=64	1.58 [0.62-4.01], p=0.338	3.07 [1.00-9.44], p=0.051
	lSUA/sCR_hTG	n=12	0.00 [0.00->100], p=0.997	0.00 [0.00->100], p=0.998
	hSUA/sCR_hTG	n=28	1.44 [0.49-4.30], p=0.509	2.87 [0.77-10.73], p=0.117
Hypertensive (n=4227)				
	lSUA/sCR_nTG	n=1639	1 (reference)	1 (reference)
	hSUA/sCR_nTG	n=1627	1.40 [1.20-1.62], p<0.001	1.22 [1.05-1.42], p=0.009
	lSUA/sCR_hTG	n=369	1.15 [0.90-1.47], p=0.254	1.61 [1.24-2.10], p<0.001
	hSUA/sCR_hTG	n=592	1.25 [1.02-1.53], p=0.032	1-52 [1.23-1.90], p<0.001
Diabetic (n=75)				
	lSUA/sCR_nTG	n=25	1 (reference)	1 (reference)
	hSUA/sCR_nTG	n=31	1.29 [0.64-2.60], p=0.476	1.15 [0.46-2.88], p=0.772
	lSUA/sCR_hTG	n=7	0.98 [0.32-3.02], p=0.977	1.84 [0.43-7.91], p=0.410
	hSUA/sCR_hTG	n=12	1.84 [0.76-4.48], p=0.179	1.91 [0.66-5.48], p=0.231
Obese and hypertensive (n=919)				
	lSUA/sCR_nTG	n=191	1 (reference)	1 (reference)
	hSUA/sCR_nTG	n=411	1.80 [1.14-2.83], p=0.011	1.38 [0.87-2.18], p=0.172
	lSUA/sCR_hTG	n=78	1.83 [0.97-3.45], p=0.061	1.89 [0.98-3.67], p=0.059
	hSUA/sCR_hTG	n=239	1.71 [1.05-2.80], p=0.032	1.81 [1.06-3.08], p=0.029
Obese and diabetic (n=18)				
	lSUA/sCR_nTG	n=3	1 (reference)	1 (reference)
	hSUA/sCR_nTG	n=6	n/a	n/a
	lSUA/sCR_hTG	n=2	n/a	n/a
	hSUA/sCR_hTG	n=7	n/a	n/a
Hypertensive and diabetic (n=524)				
	lSUA/sCR_nTG	n=159	1 (reference)	1 (reference)
	hSUA/sCR_nTG	n=187	1.11 [0.81-1.52], p=0.521	1.12 [0.80-1.56], p=0.511
	lSUA/sCR_hTG	n=77	1.14 [0.77-1.71], p=0.514	1.51 [0.97-2.35], p=0.069
	hSUA/sCR_hTG	n=101	1.16 [0.81-1.68], p=0.412	1.58 [1.03-2.43], p=0.035
Obese, hypertensive and diabetic (n=257)				
	lSUA/sCR_nTG	n=54	1 (reference)	1 (reference)
	hSUA/sCR_nTG	n=93	1.33 [0.79-2.24], p=0.290	1.44 [0.83-2.49], p=0.190
	lSUA/sCR_hTG	n=31	1.20 [0.60-2.40], p=0.600	1.00 [0.49-2.07], p=0.993
	hSUA/sCR_hTG	n=79	1.39 [0.81-2.40], p=0.232	1.31 [0.73-2.35], p=0.357

Hazard ratios [95% confidence interval] for SUA/serum creatinine and TG cut-offs in univariable and multivariable analysis for all-cause mortality across the cardiometabolic spectrum. Age, sex, BMI, the active consumption of alcohol, the current smoking habit, SBP, DBP, total cholesterol, HDL, glycaemia, hematocrit and diuretics were used as confounders. Data were analysed by Cox regression analysis. The analysis was not performed on people with obesity and type diabetes and no other comorbidities due to the small sample size of the group (n=18). Data are reported for completeness in undersized groups (n<50). p <0.05 was considered statistically significant *>100: hazard ratio or confidence interval value higher than 100. lSUA/sCR_hTG: low SUA/serum creatinine and hypertriglyceridemia TG. lSUA/sCr_nTG: low SUA/serum creatinine and normotriglyceridemia. hSUA/sCr_hTG: high SUA/serum creatinine and hypertriglyceridemia TG. hSUA/sCr_nTG: high SUA/serum creatinine and normotriglyceridemia. TG: triglycerides. SUA: serum uric acid.*

Supplementary Table S8. Exploration of cardiovascular mortality across the cardiometabolic spectrum of combined SUA and TG strata adopting SUA/serum creatinine>5.35 and TG≥150 mg/dL as cut-offs.

**All-cause mortality**				
			Univariable	Multivariable
Healthy (n=1956)				
	lSUA/sCR_nTG	n=863	1 (reference)	1 (reference)
	hSUA/sCR_nTG	n=862	1.29 [0.81-2.07], p=0.284	1.37 [0.84-2.24], p=0.210
	lSUA/sCR_hTG	n=151	0.33 [0.04-2.40], p=0.273	0.22 [0.03-1.65], p=0.140
	hSUA/sCR_hTG	n=80	1.67 [0.80-3.49], p=0.173	1.34 [0.59-3.04], p=0.486
Obese (n=148)				
	lSUA/sCR_nTG	n=44	1 (reference)	1 (reference)
	hSUA/sCR_nTG	n=64	2.58 [0.52-12.81], p=0.245	7.59 [0.88-65.10], p=0.065
	lSUA/sCR_hTG	n=12	0.00 [0.00->100], p=0.998	0.00 [0.00->100], p=0.999
	hSUA/sCR_hTG	n=28	1.63 [0.23-11.60], p=0.624	4.05 [0.29-56.84], p=0.299
Hypertensive (n=4227)				
	lSUA/sCR_nTG	n=1639	1 (reference)	1 (reference)
	hSUA/sCR_nTG	n=1627	1.40 [1.13-1.73], p=0.002	1.18 [0.94-1.46], p=0.148
	lSUA/sCR_hTG	n=369	1.23 [0.87-1.75], p=0.241	1.53 [1.05-2.24], p=0.027
	hSUA/sCR_hTG	n=592	1.67 [0.80-3.49], p=0.173	1.41 [1.03-1.92], p=0.032
Diabetic (n=75)				
	lSUA/sCR_nTG	n=25	1 (reference)	1 (reference)
	hSUA/sCR_nTG	n=31	1.09 [0.43-2.77], p=0.860	0.72 [0.19-2.80], p=0.638
	lSUA/sCR_hTG	n=7	1.20 [0.32-4.51], p=0.792	4.95 [0.68-36.15], p=0.115
	hSUA/sCR_hTG	n=12	1.62 [0.48-5.45], p=0.438	1.48 [0.32-6.84], p=0.619
Obese and hypertensive (n=919)				
	lSUA/sCR_nTG	n=191	1 (reference)	1 (reference)
	hSUA/sCR_nTG	n=411	1.84 [0.94-3.58], p=0.075	1.29 [0.65-2.55], p=0.460
	lSUA/sCR_hTG	n=78	1.50 [0.56-4.07], p=0.421	1.83 [0.65-5.20], p=0.254
	hSUA/sCR_hTG	n=239	1.67 [0.81-3.47], p=0.167	2.19 [0.98-4.85], p=0.055
Obese and diabetic (n=18)				
	lSUA/sCR_nTG	n=3	1 (reference)	1 (reference)
	hSUA/sCR_nTG	n=6	n/a	n/a
	lSUA/sCR_hTG	n=2	n/a	n/a
	hSUA/sCR_hTG	n=7	n/a	n/a
Hypertensive and diabetic (n=524)				
	lSUA/sCR_nTG	n=159	1 (reference)	1 (reference)
	hSUA/sCR_nTG	n=187	1.24 [0.82-1.88], p=0.314	1.22 [0.78-1.89], p=0.379
	lSUA/sCR_hTG	n=77	1.12 [0.65-1.95], p=0.679	1.33 [0.72-2.43], p=0.362
	hSUA/sCR_hTG	n=101	1.33 [0.83-2.15], p=0.240	1.67 [0.95-2.93], p=0.074
Obese, hypertensive and diabetic (n=257)				
	lSUA/sCR_nTG	n=54	1 (reference)	1 (reference)
	hSUA/sCR_nTG	n=93	1.43 [0.72-2.84], p=0.304	1.82 [0.88-3.77], p=0.109
	lSUA/sCR_hTG	n=31	1.30 [0.53-3.19], p=0.562	1.44 [0.44-2.95], p=0.789
	hSUA/sCR_hTG	n=79	1.24 [0.60-2.59], p=0.561	1.39 [0.63-3.07], p=0.441

Hazard ratios [95% confidence interval] for SUA/serum creatinine and TG cut-offs in univariable and multivariable analysis for cardiovascular mortality across the cardiometabolic spectrum. Age, sex, BMI, the active consumption of alcohol, the current smoking habit, SBP, DBP, total cholesterol, HDL, glycaemia, hematocrit and diuretics were used as confounders. Data were analysed by Cox regression analysis. The analysis was not performed on people with obesity and type diabetes and no other comorbidities due to the small sample size of the group (n=18). Data are reported for completeness in undersized groups (n<50). $p < 0.05$ was considered statistically significant *>100: hazard ratio or confidence interval value higher than 100. lSUA/sCR_hTG: low SUA/serum creatinine and hypertriglyceridemia TG. lSUA/sCr_nTG: low SUA/serum creatinine and normotriglyceridemia. hSUA/sCr_hTG: high SUA/serum creatinine and hypertriglyceridemia TG. hSUA/sCr_nTG: high SUA/serum creatinine and normotriglyceridemia. TG: triglycerides. SUA: serum uric acid.*

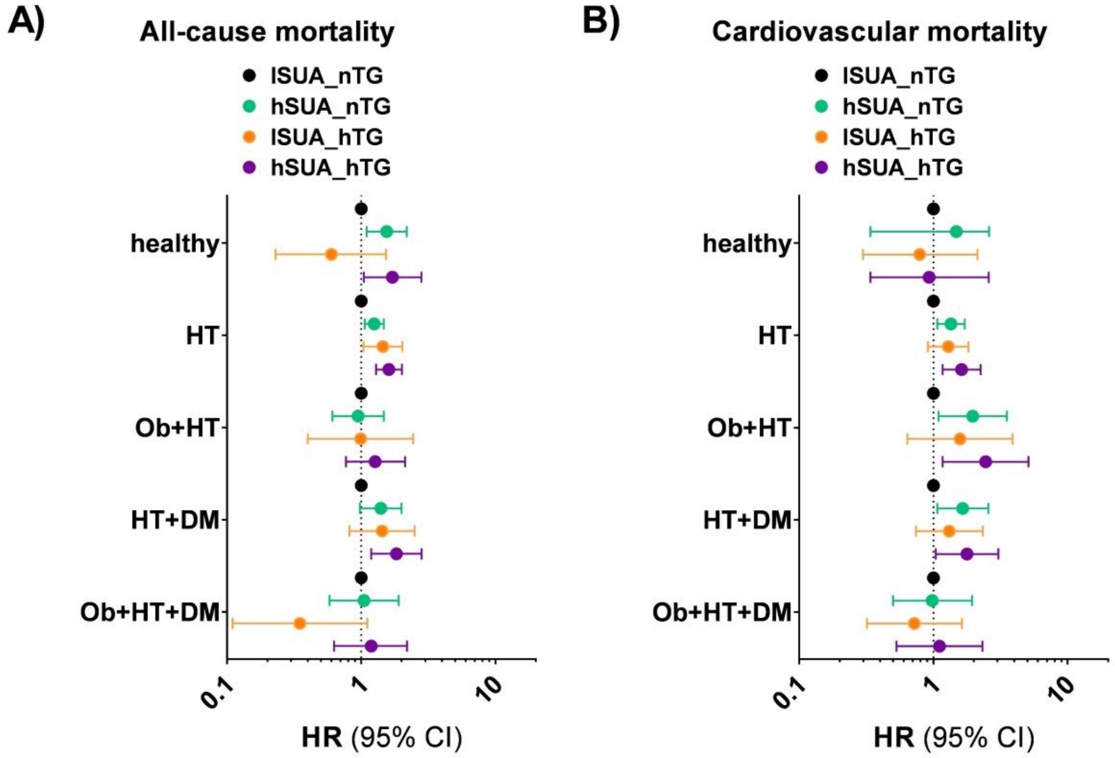

Supplementary Figure S1. Trends for all-cause mortality (A) and cardiovascular mortality (B) across cardiometabolic spectrum in patients across combined SUA and TG strata, adopting SUA≥4.7 mg/dL and SUA≥5.6 mg/dL as cut-offs for all-cause mortality and cardiovascular mortality, respectively. Low SUA and normotriglyceridemia TG (black dots and lines); low SUA and hypertriglyceridemia (green dots and lines); high SUA and normotriglyceridemia TG (orange dots and lines); high SUA and hypertriglyceridemia (violet dots and lines). The analysis was not run on people with obesity and type diabetes and no other comorbidities due to the small sample size of the subgroup (n=18). Data in undersized groups (n<50) are reported for completeness in Supplementary Tables 3-4. *CI: confidence interval. DM: patients with diabetes and no other comorbidities. HR: hazard ratio. HT: patients with hypertension and no other comorbidities. HT+DM: patients with hypertension and diabetes and no other comorbidities; hSUA_hTG: high SUA and hypertriglyceridemia TG. hSUA_nTG: high SUA and normotriglyceridemia. lSUA_hTG: low SUA and hypertriglyceridemia TG. lSUA_nTG: low SUA and normotriglyceridemia. Ob: patients with obesity and no other comorbidities; Ob+HT: patients with obesity and hypertension and no other comorbidities; Ob+HT+DM: patients with obesity, hypertension and diabetes and no other comorbidities.*
